# Intralobar pulmonary sequestration complicated by a giant supplying artery aneurysm presenting for 9 years: a case report

**DOI:** 10.3389/fsurg.2025.1481819

**Published:** 2025-02-04

**Authors:** Wei Weng, Xinle Chi, Ze Liu, Weiqian Chen, Shengze Wang, Wenjie Cai, Hai Wu, Yaomeng Chen

**Affiliations:** ^1^ Department of Radiology, The Wenzhou Third Clinical Institute Affiliated to Wenzhou Medical University, Wenzhou People’s Hospital, Wenzhou, Zhejiang, China; ^2^ Department of Nuclear Medicine, Ningbo Hangzhou Bay Hospital, Ningbo, Zhejiang, China; ^3^ Department of Respiratory Medicine, The Wenzhou Third Clinical Institute Affiliated to Wenzhou Medical University, Wenzhou People’s Hospital, Wenzhou, Zhejiang, China; ^4^ Department of Science and Education, The Wenzhou Third Clinical Institute Affiliated to Wenzhou Medical University, Wenzhou People’s Hospital, Wenzhou, Zhejiang, China

**Keywords:** intralobar pulmonary sequestration, aneurysm, thoracoscope, congenital anomaly, case report

## Abstract

**Background:**

This article reports a case of a intralobar pulmonary sequestration (IPS) with a significantly large feeding artery aneurysm and shares the successful treatment experience.

**Case presentation:**

A male was diagnosed with IPS combined with a feeding artery aneurysm approximately 74 mm × 61 mm in nine years ago but refused thoracotomy at that time. The patient presented this time due to an infection, and a CT scan revealed a significant increase in the lesion size to approximately 123 mm × 100 mm. After controlling the pulmonary inflammation, the patient underwent thoracoscopic ligation of the supplying artery, followed by an open chest excision of the sequestrated pulmonary aneurysm and left lower lobectomy, and the recovery post-surgery was very good.

**Conclusion:**

When a sequestrated lung enlarges significantly, it is crucial to consider not only the conventional possibility of malignancy but also the rare coexistence of aneurysms. Thoracoscopic ligation of the supplying artery followed by open chest surgery is an effective treatment approach for IPS combined with a large feeding artery aneurysm.

## 1 Introduction

Intralobar pulmonary sequestration (IPS) is a rare congenital lung tissue developmental anomaly where a non-functional lung tissue mass exists independently outside of normal lung tissue (1). The sequestrated lung is separated from the normal airway bronchial tree and pulmonary artery, with its blood supply originating from the systemic circulation, primarily from the descending aorta (2). An arterial aneurysm refers to a sustained and localized enlargement of an artery, exhibiting a diameter that is at least 50% greater than that of the adjacent, unaffected segment of the vessel (3). Most aneurysms are asymptomatic; however, they can rupture due to factors such as trauma, emotional stress, or hypertension, leading to a high mortality rate once symptoms appear (4–6).

The combination of IPS and a feeding artery aneurysm is rare and is typically treated surgically as soon as it is discovered. However, this patient delayed surgery for 9 years, which allowed us to observe the progression of this disease. For similar cases, the main reported treatment approaches include thoracotomy (7) and hybrid surgery that consists of thoracic endovascular aortic repair (TEVAR) combined with IPS resection and left lower lobectomy via video-assisted thoracoscopic surgery (VATS) (8, 9). Reports on such cases are rare, and there are currently no guideline-based treatment methods. This case was treated without the implantation of a stent, which avoids the long-term use of anticoagulants after stent placement. Additionally, this case report provides specific information on the surgical approach and the patient's prognosis, offering an effective reference for the treatment of similar conditions.

## 2 Case report

**Figure 1:**
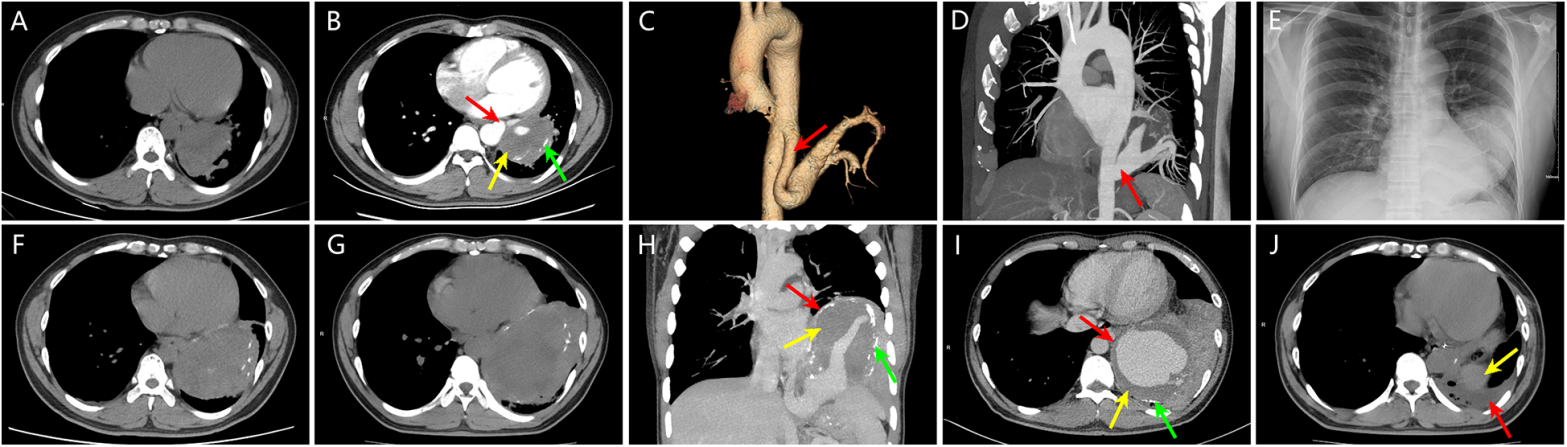
**(A)** Nine years ago, chest CT revealed a mass in the left lower lobe measuring approximately 74 mm × 61 mm. **(B)** Nine years ago, contrast-enhanced chest CT showed a clearly defined feeding artery (red arrow) supplying the left lower lobe mass, with a maximum diameter of approximately 65 mm × 43 mm, a normal proximal diameter of about 12 mm, indicating vascular dilation of at least 3.6 times; mural thrombosis within the vascular lumen (yellow arrow) and calcified plaques in the vessel wall (green arrow) were also observed. **(C, D)** Nine years ago, post-aortic angiography volume rendering (VR) and maximum intensity projection (MIP) images clearly depicted the feeding artery originating from the thoracic aorta (red arrow), confirming the diagnosis of intralobar pulmonary sequestration complicated by a supplying artery aneurysm. **(E, F)** One year ago, chest x-ray and CT showed a left lower lobe mass measuring approximately 112 mm × 82 mm, indicating significant enlargement compared to nine years earlier. **(G)** At this visit, chest CT revealed the left lower lobe mass measuring approximately 137 mm × 96 mm, further increasing in size compared to one year ago. **(H, I)** At this visit, contrast-enhanced chest CT and MIP images displayed a giant aneurysm of the sequestrated lung and its supplying artery (red arrow), with the aneurysm's maximum diameter measuring approximately 123 mm × 100 mm, with a normal proximal diameter of about 13 mm, indicating vascular dilation of at least 7.7 times. Mural thrombosis within the vascular lumen (yellow arrow) and calcified plaques in the vessel wall (green arrow) were also visible. **(J)** One month post-surgery, follow-up chest CT revealed small pleural effusion (red arrow), with the yellow arrow indicating the spleen.

A 38-year-old male was admitted due to “coughing with sputum for over 3 months and fever for more than 10 days”. Nine years ago, he was diagnosed with a left lower lobe mass, measuring approximately 74 mm × 61 mm, after a chest CT revealed lung infection (Figure 1A). Contrast-enhanced CT (Figure 1B) and aortography (Figures 1C,D) indicated the mass had a feeding artery originating from the thoracic aorta, with a thick mural thrombus and calcified plaques in the arterial wall. The widest part of the feeding artery measured about 65 mm × 43 mm, with a normal proximal diameter of approximately 12 mm, indicating vascular dilation of at least 3.6 times, which confirmed the diagnosis of IPS complicated by feeding artery aneurysm. Although doctors recommended thoracotomy, the patient refused. One year ago, the patient sought treatment for a lung infection, with a chest x-ray and CT scan (Figures 1E,F) showing the left lower lobe mass enlarged to approximately 112 mm × 82 mm. The patient again refused thoracotomy. Three months before this admission, he developed severe coughing and sputum production, along with fever and generalized muscle aches 10 days prior. Upon admission, his temperature was 38.8°C, dullness on percussion was noted in the left lower lung, and breath sounds were diminished. Laboratory findings showed a white blood cell count of 10.31 × 10^9^/L (normal range: 4.0–10.0 × 10^9^/L), C-reactive protein at 233.28 mg/L (normal range <2.87 mg/L). CT results (Figure 1G) indicated the mass further enlarged to approximately 137 mm × 96 mm compared to one year prior. Enhanced CT and MIP images (Figures 1H,I) showed the aneurysm's widest part measuring approximately 123 mm × 100 mm, with a normal proximal diameter around 13 mm, indicating vascular dilation of at least 7.7 times, along with a thick mural thrombus and calcified plaques. After the pulmonary infection was treated, the doctor advised the patient to undergo surgery as soon as possible to avoid life-threatening complications, and the patient ultimately consented to the surgical procedure. The doctor recommended that he undergo TEVAR combined with IPS resection and left lower lobectomy via VATS. However, he did not want to have a stent implanted, as he wished to avoid long-term or even lifelong anticoagulation therapy; therefore, the final surgical plan did not include stent implantation. The specific surgical steps are as follows: After cannulation of the femoral artery and vein to establish extracorporeal circulation, access to the thoracic cavity is obtained through the left posterior lateral sixth intercostal space. Under thoracoscopic guidance, the aorta is dissected at the T6 and T12 levels, followed by ligation of the supplying artery. Subsequently, the thoracic surgeon performs a left lower lobectomy and concurrently excises the aneurysm. One month post-operation, a follow-up CT (Figure 1J) revealed complete removal of the left lower lobe mass, with a small amount of pleural effusion observed, and the patient was recovering well.

## 3 Discussion

The prevailing theory concerning the development of pulmonary sequestration suggests that it arises from the formation of an accessory lung bud located posterior to the normal lung buds (2). Aneurysms frequently arise from cystic medial degeneration, which weakens the arterial walls (10). At the time of this visit, the size of the aneurysm was approximately 123 mm × 100 mm. As a branch artery of the thoracic aorta, this size is striking, even compared to an aortic aneurysm. Reports by Ryan et al. indicate that when the diameter of a thoracic aortic aneurysm exceeds 6 cm, the rates of death, rupture, or dissection can be as high as 15.6% (11). John et al. noted that the risk of aortic rupture or dissection sharply increases when the descending aorta's diameter reaches 6.5 cm (12). However, in this case, despite the aneurysm being so large, it remained stable for nine years without dissection or rupture, likely due to the presence of a thick mural thrombus within the aneurysm cavity and its protective effect against rupture (13). The blood flow prone to turbulence in the feeding artery of the sequestrated lung, providing a hemodynamic basis for thrombus formation (14, 15). Additionally, we speculate that the external sequestrated lung may also act as an “extra arterial wall”, somewhat limiting the expansion of the aneurysm. The mural thrombus and the sequestrated lung improve the feeding artery's capacity to withstand high pressure and compensate. Nonetheless, the aneurysm continues to grow and will eventually rupture, leading to mortality. Thus, the patient is both unfortunate and fortunate. Although IPS poses a health risk, the sequestrated lung and the mural thrombus serve as protective factors that limit the rupture of the aneurysm. This reflects the interbalancing, multifaceted, and complex nature of diseases, indicating that the perceptions of good and bad in relation to diseases are not fixed. The clinical thoracoscopic combined open surgery excision of the patient's lesion was highly successful. Compared to a smaller sequestrated lung lesion associated with a feeding artery aneurysm (103 mm × 72 mm) (8), this patient did not require the placement of a stent. This allows patients to avoid the need for long-term or lifelong anticoagulation therapy after surgery, making it a better treatment option for them, which provides insights for the treatment of similar cases in the future.

## 4 Conclusion

When a IPS shows progressive enlargement, it is vital to consider not only the possibility of malignancy but also the presence of aneurysm formation and its growth in the supplying artery. We can avoid the need for a stent as much as possible by treating the disease through thoracoscopic ligation of the supplying artery and open surgery for lesion excision, thus sparing the patient from the long-term or even lifelong use of anticoagulants associated with stent implantation.

## Data Availability

The original contributions presented in the study are included in the article/Supplementary Material, further inquiries can be directed to the corresponding author.
